# Alcohol brief intervention for hospitalized veterans with hazardous drinking: protocol for a 3-arm randomized controlled efficacy trial

**DOI:** 10.1186/s13722-015-0033-6

**Published:** 2015-05-13

**Authors:** Lauren M Broyles, Melissa E Wieland, Andrea L Confer, Monica M DiNardo, Kevin L Kraemer, Barbara H Hanusa, Ada O Youk, Adam J Gordon, Mary Ann Sevick

**Affiliations:** 1 Center for Health Equity Research and Promotion, Veterans Affairs (VA) Pittsburgh Healthcare System, University Drive C (151C), Pittsburgh, PA 15240 USA; 2 Division of General Internal Medicine, Department of Medicine, School of Medicine, University of Pittsburgh, 230 McKee Place, Suite 600, Pittsburgh, PA 15213 USA; 3 Veterans Integrated Service Network 4 (VISN4) Mental Illness Research, Education, and Clinical Center, VA Pittsburgh Healthcare System, University Drive C (151C), Pittsburgh, PA 15240 USA; 4 Department of Biostatistics, Graduate School of Public Health, University of Pittsburgh, Pittsburgh, PA USA; 5 Department of Population Health, Center for Healthful Behavior Change, New York University School of Medicine, 227 East 30th Street, New York, NY 10016 USA

**Keywords:** Inpatients, Risk reduction behavior, Drinking behavior, Alcohol drinking, Binge drinking, Brief intervention, Clinical trial, Assessment reactivity, Measurement effects

## Abstract

**Background:**

Various hospital accreditation and quality assurance entities in the United States have approved and endorsed performance measures promoting alcohol brief intervention (BI) for hospitalized individuals who screen positive for unhealthy alcohol use, the spectrum of use ranging from hazardous use to alcohol use disorders. These performance measures have been controversial due to the limited and equivocal evidence for the efficacy of BI among hospitalized individuals. The few BI trials conducted with hospital inpatients vary widely in methodological quality. While the majority of these studies indicate limited to no effects of BI in this population, none have been designed to account for the most pervasive methodological issue in BI studies presumed to drive study findings towards the null: assessment reactivity (AR).

**Methods/Design:**

This is a three-arm, single-site, randomized controlled trial of BI for hospitalized patients at a large academic medical center affiliated with the U.S. Department of Veterans Affairs who use alcohol at hazardous levels but do not have an alcohol use disorder. Participants are randomized to one of three study conditions. Study Arm 1 receives a three-part alcohol BI. Study Arm 2 receives attention control. To account for potential AR, Study Arm 3 receives AC with limited assessment. Primary outcomes will include the number of standard drinks/week and binge drinking episodes reported in the 30-day period prior to a final measurement visit obtained 6 months after hospital discharge. Additional outcomes will include readiness to change drinking behavior and number of adverse consequences of alcohol use. To assess differences in primary outcomes across the three arms, we will use mixed-effects regression models that account for a patient’s repeated measures over the timepoints and clustering within medical units. Intervention implementation will be assessed by: a) review of intervention audio recordings to characterize barriers to intervention fidelity; and b) feasibility of participant recruitment, enrollment, and follow-up.

**Discussion:**

The results of this methodologically rigorous trial will provide greater justification for or against the use of BI performance measures in the inpatient setting and inform organizational responses to BI-related hospital accreditation and performance measures.

**Trial registration:**

NCT01602172

## Background

Unhealthy alcohol use comprises the spectrum of alcohol use, ranging from hazardous drinking, (defined as consumption that exceeds 14 standard drinks per week or 4 per occasion for men, and 7 standard drinks per week or 3 per occasion for women and healthy individuals age 65 or older) to alcohol use disorders [1-3]. In 2008, The Joint Commission (TJC), a hospital accreditation body in the United States, began the development and testing of a set of standardized hospital performance measures to address the entire spectrum of alcohol use through screening, brief intervention (BI) counseling, referral to specialty treatment, pharmacotherapy, and follow-up for hospitalized patients [4]. Because the measures are designed to be applicable to all hospitalized patients, regardless of reason for admission, they are categorized as global performance measures. The measures are not mandatory, but in January 2012, they began being offered as one of the sets that hospitals could select to achieve in order to receive ongoing accreditation [5]. In February 2014, the measures were endorsed by the U.S. National Quality Forum [6]. However, during their initial development and release, the measures generated considerable debate [7-11]. Opponents of the measures expressed concerns regarding the limited and equivocal nature of the evidence for efficacy of BI among hospitalized individuals. [7,8,12,13]. Meanwhile, proponents of the measures endorsed their uptake and implementation, challenging the need for continued alcohol BI trials across different health-care settings for patients with varying levels of alcohol involvement and for different combinations of substances [11].

At the time of this debate, few trials of alcohol BI had even been conducted in hospitalized patients [12,14-16], with only one of these conducted in a sample of U.S. drinkers [12]. Because cultural norms and attitudes about alcohol use vary internationally, caution must be exercised in generalizing the results of BI trials from outside the United States. Additionally, while the methodological quality of these BI studies varies widely, the majority did not demonstrate significant reductions in alcohol consumption or in the adverse consequences of alcohol use [12,14,16]. Subsequent post-hoc analyses, however, suggested that BI was in fact efficacious for decreasing alcohol consumption at 3 months among nondependent, hazardous drinkers (i.e., patients whose use met criteria for hazardous drinking but did not simultaneously meet criteria for alcohol dependence) [13]. Additionally, patients consuming alcohol at nondependent, hazardous levels who had an alcohol-attributable admitting diagnosis had significantly fewer heavy episodic (binge) drinking days at 3-month follow-up [17].

Since this active debate, three additional trials of BI for hospitalized patients have been conducted outside the United States, but again, with equivocal results and/or coarser outcomes, such as change in alcohol screening status, as opposed to more specific measures of alcohol consumption [18-20]. BI researchers have long suggested that null or inconsistent findings in their trials are attributable to the fact that control groups in these studies regularly showed decreased alcohol consumption, thus reducing the ability to demonstrate BI’s effects [21,22]. Furthermore, these reductions in control group drinking have consistently been explained by assessment reactivity (AR)—the potential for extensive and/or repeated study assessments of alcohol use to mimic the components of alcohol BI. When AR occurs, study results can be biased towards the null because in essence, both study groups inadvertently receive some degree of intervention [21-23]. Despite the frequency with which BI investigators ascribe null findings to AR, few researchers have designed BI trials to take its potential effects into account [21,24-26], and none have sought to do so in trials of hospitalized individuals.

Our overarching goal is to inform the debate about the efficacy of alcohol BI for hospitalized individuals in a methodologically rigorous trial using a U.S.-based sample of hazardous drinkers. Furthermore, our study design addresses several key methodological issues that have been raised as concerns regarding other BI trials. To address (or avoid) AR, we employ a three-arm randomized design: Arm 1 consists of veterans randomized to BI (BI); Arm 2 consists of veterans randomized to Attention Control (AC); and Arm 3 consists of veterans randomized to an AC group with limited assessment (AC-LA) (i.e., alcohol consumption variables only).

### Specific aims and hypotheses

The Specific Aims of this three-arm randomized controlled trial (RCT) are to: (1) determine the impact of BI on the alcohol screening status, number of drinks/week, number of binge drinking episodes, readiness to change drinking behavior, and adverse consequences of alcohol use in hospitalized hazardous drinkers; and (2) evaluate the process of intervention implementation by characterizing barriers to intervention fidelity and assessing the feasibility of recruitment, enrollment, and follow-up of hospitalized hazardous drinkers.

We hypothesize that at 6 months post-discharge, Arm 1 (BI) will report greater reductions in the alcohol consumption outcomes than Arms 2 (AC) and 3 (AC-LA). Furthermore, we hypothesize that AR will result in some reductions in alcohol consumption in Arm 2, and that the reductions in Arm 2 will be greater than those displayed in Arm 3, but less than those displayed by Arm 1.

## Methods

### Human subjects protections

This study is approved by the Research and Development Service and the Institutional Review Board (IRB) of the U.S. Department of Veterans Affairs (VA) Pittsburgh Healthcare System (VAPHS).

### Design

This study is a three-arm, single-site, RCT of BI for hospitalized, hazardous drinkers. Participants are randomized to one of three study conditions: Arm 1: a group receiving a 3-part alcohol BI; Arm 2: an AC group; and Arm 3: an AC-LA group.

### Setting and participants

This study is being conducted on the three medical-surgical (Med-Surg) units at the University Drive campus of the VAPHS, a large academic medical center in southwestern Pennsylvania that is part of the VA. The University Drive campus is home to a 146-bed hospital facility that provides routine medical, neurological, psychiatric, and surgical care in addition to specialized services. Patient and unit characteristics for the three participating Med-Surg units are provided in Table 1.

**Table 1:** **Medical-surgical unit and patient profile, VAPHS, FY 2010**

**Parameter type of unit total for 3 units**				
		**Medical**	**Surgical**	**Total**
Admissions/month		407	240	647
Average daily census		67	49	58
Average length of stay (days)	Unit A	Unit B	Unit C	3.9
	(Surg)	(Med-Tele)	(Med-Liver)	
	3.6	3.1	5.0	
Female veterans (%)				4
OEF/OIF veterans (%)				2
Average patient age				65
% Age 50 or under				10

Note: Med = Medical; Liver = Liver Transplant; Surg = Surgical; Tele = Telemetry; VAPHS = VA Pittsburgh Healthcare System.

Patient inclusion criteria for the study are: admission to a participating unit; age 21 or older; and hazardous, nondependent alcohol consumption (defined in Screening section below). Study exclusion criteria are: participation in alcohol treatment and/or 12-step programs in the previous 6 months; current drug or alcohol dependence; significant cognitive impairment; diagnosis of a bipolar or psychotic disorder; residence in a restricted housing setting (e.g., skilled nursing facility); lack of telephone access or unwillingness to be contacted for follow-up; sensory impairment precluding communication; and medically-related inability to participate or consent to study participation.

### Recruitment, screening, and enrollment

**Figure 1:**
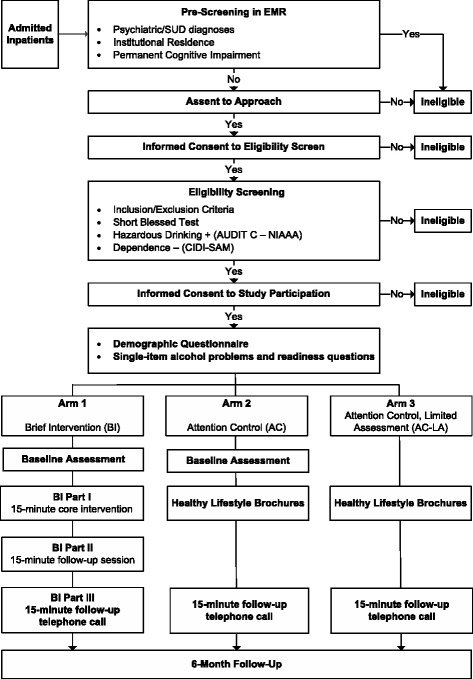
Study design and procedures.

In the inpatient care setting, the logistics of screening and recruitment of hospitalized patients for research study participation require careful consideration. Adherence to federal and local human subjects protections for research can be challenged by patient acuity, inpatient processes of care (e.g., medication administration/side effects, bedside and off-unit procedures), and the need for countless assessments by professionals from multiple disciplines and sub-specialties. Our multi-step procedures for prescreening, approach, screening, informed consent, and study enrollment are described below and depicted in Figure 1.

### Prescreening

Because of the high volume of admissions to the participating units (approximately 650 admissions each month), we have a Waiver of Informed Consent from the IRB at VAPHS that authorizes us to generate a daily list of patients admitted to the participating units and allows for review of each admitted patient’s electronic medical record (EMR) in order to identify ineligibility criteria (e.g., housing status, psychiatric diagnosis). This prescreening process allows us to avoid exerting unnecessary burden on acutely ill patients who could be determined ineligible by record review, and reduces the volume of patients to be approached for interest and screening. After review of the EMR, a list of patients who remain eligible for the study is prepared for each unit.

### Screening

According to VAPHS IRB regulations, patients may not be directly approached about research participation by individuals who are not directly associated with the patient’s care; a clinical care provider must first secure permission for a member of the research staff to approach the patient. For this reason, our study’s Research Assistant (RA) first visits each of the three participating units and presents the charge nurse with the list of the prescreened patients admitted to that unit. The charge nurse (or delegate) then approaches each patient on the list to request his/her permission to allow the RA to approach the patient (i.e., enter the room to provide a study overview and begin the recruitment process). The RA then provides the patient with a general description of the study. If interested, the patient is asked to provide verbal consent for eligibility screening, which is performed by the RA. In Table 2, the four-step study screening process is represented by the “Eligibility Screening (ES)” column for each study arm.

**Table 2:** **Study assessments by study arm and timepoint**

		**Arm 1 brief intervention (BI)**				**Arm 2 attention control (AC)**				**Arm 3 attention control, limited assessment (AC-LA)**			
		**Timepoint**				**Timepoint**				**Timepoint**			
**Variable**	**Instrument**	**ES**	**PC**	**B**	**FU**	**ES**	**PC**	**B**	**FU**	**ES**	**PC**	**B**	**FU**
Cognitive status	Short Blessed Test	X				X				X			
Alcohol screening status	AUDIT-C	X			X	X			X	X			X
Drinks per week and Binge episodes last 30 days	3 NIAAA questions	X			X	X			X	X			X
Alcohol dependence	Modified CIDI-SAM	X				X				X			
Sociodemographic/ clinical data	Sociodemographic form		X		X		X		X		X		X
Readiness to change drinking behavior and adverse consequences of alcohol use	*Single items* from SOCRATES and SIP-2R										X		
Readiness to change drinking behavior	SOCRATES			X	X			X	X				X
Adverse consequences of alcohol use	SIP-2R			X	X			X	X				X
Participant responsiveness (patient opinions about the intervention)	4-item investigator developed instrument			X									

**Note:** AUDIT-C = Alcohol Use Disorders Identification Test, Consumption; NIAAA = National Institute on Alcohol Abuse and Alcoholism; SOCRATES = Stages of Change Readiness and Treatment Eagerness Scale; SIP-2R = Short Inventory of Problems; **Timepoints** – ES = Eligibility Screening (10–25 minutes); PC = Post Consent (5 minutes); B = Baseline (10 minutes); FU = Follow-up at 6 months (30–45 minutes).

Because hospitalized individuals may have altered mental status due to acute illness or receipt of sedating medication, the Short Blessed Test (SBT) is first performed to assess for cognitive impairment. The SBT is a 6-item screening instrument used to identify cognitive deficits in orientation, registration, and attention [27]. Patients are then assessed for hazardous alcohol consumption using criteria from the U.S. National Institute on Alcohol Abuse and Alcoholism (NIAAA). Individuals who consume more than 14 drinks/week or 4 drinks/occasion (men); or more than 7 drinks/week or 3 drinks/occasion (women) are considered hazardous drinkers [2]. Number of drinks consumed per week is determined by the product of responses to the following two questions: (1) *On average, how many days a week do you have an alcoholic drink?* and (2) *On a typical drinking day, how many standard-sized drinks do you have?* [28]. A “standard-sized drink” refers to 12 ounces of beer, 5 ounces of wine, or 1.5 ounces of liquor/spirits [2]. Number of binge drinking episodes is assessed through a third question: *How many times in the past 30 days have you had 5 or more standard-sized drinks in a day* (men), *or 4 or more standard-sized drinks in a day?* (women) [2]. The use of NIAAA criteria to determine the need for an alcohol BI provides us with precise quantification of alcohol use for measurement of our primary outcome (number of drinks/week) and is consistent with VA/Department of Defense Clinical Practice Guidelines, which recommend BI for individuals who drink above the specified limits or who drink despite contraindications [28].

Potential participants also complete the Alcohol Use Disorders Identification Test – Consumption (AUDIT-C) to provide an additional alcohol outcome measure that is consistent with and easily comparable to clinical practice in VA primary care and TJC measure specifications. The AUDIT-C is a widely used and validated three-item alcohol screen that can help identify persons who are hazardous drinkers. The AUDIT-C is scored on a scale of 0–12. For men, total scores of 4 or more are considered positive; for women, scores of 3 or more are considered positive [29,30].

Section C (Alcohol) from the Composite International Diagnostic Interview, Substance Abuse Module (CIDI-SAM) [31] is administered to rule out hazardous drinkers who also meet criteria for alcohol dependence (Table 2). The CIDI-SAM is a comprehensive, fully structured diagnostic interview for the assessment of mental disorders that provides, by means of algorithms, lifetime and current diagnoses according to the accepted definitions of ICD-10 and the Diagnostic and Statistical Manual of Mental Disorders, Fourth Edition (DSM-IV) [31,32]. Section C of the CIDI-SAM can be administered by trained clinician or nonclinician interviewers in approximately 15 minutes in general populations outside of psychiatric treatment settings [31]. In partnership with the instrument developers, we modified Section C of the CIDI-SAM to facilitate its use in the specific context of this research study (for the rapid identification of potential alcohol dependence to determine study eligibility). All staff were trained by the instrument developers to administer this modified instrument, which contained items C16–C18, C20, C25–C30, C33, and CARD 7 of the original CIDI-SAM.

Hazardous drinkers who do not meet criteria for alcohol dependence according to CIDI-SAM are eligible for study participation. Eligible individuals then complete the informed consent process with the RA. While the RA then determines the patient’s randomization assignment, each patient completes a basic investigator-developed sociodemographic and clinical information form. General health behavior questions extracted directly from the Behavioral Risk Factor Surveillance System questionnaire are embedded within the sociodemographic form to assess other health behaviors such as tobacco use, physical activity, and diet [31,33].

### Randomization

Participants are randomized to one of the three study conditions (arms) described below. Randomization assignments were generated by the study statistician prior to study start and placed in masked envelopes. Randomization sequences were stratified by sex, race, and hospital unit in a 2:1:1 ratio. This unequal allocation to the three study conditions allows more veterans to be exposed to the expected efficacious intervention and more observations to be available for analyses of mediators and moderators of positive outcomes in the BI group.

### Arm 1: Brief intervention

Arm 1 comprises the experimental BI condition (Table 3). The three-part BI is adapted from the Brief Negotiated Interview [34] and supplemented with alcohol-related patient education materials available through the VA [35,36] and NIAAA [37]. Part I, the core BI, consists of a multi-component motivational discussion with the nurse interventionist within 12 hours of baseline assessment, which includes personalized risk feedback, advice to abstain or reduce consumption, and the negotiation of an individual change plan [28,34,38,39]. Part II consists of a single, brief, in-hospital or telephone follow-up session, with the same nurse interventionist, to briefly reinforce the original intervention, discuss and “troubleshoot” the patient’s change plan, and offer additional encouragement. Part III of the intervention consists of a brief follow-up telephone call 2 weeks following hospital discharge, during which the same nurse interventionist again briefly reinforces the original intervention, discusses the patient’s change plan, and offers additional encouragement (Table 3). Typical BI follow-up periods for hospitalized patients range from one week (for written materials) to one month (for additional intervention) [12,40]. We selected a 2-week period in order to verbally reinforce recent intervention material, while allowing recently hospitalized patients time to readjust and resume self-care practices. For patients who do not initially establish an alcohol consumption goal or initiate a change plan during the initial BI, the follow-up session focuses on exploring and supporting general readiness to change.

**Table 3:** **Components of 3-part brief intervention for hospitalized hazardous drinkers**

**Part I: Core brief intervention (~15 minutes)** [28,34,38,39]	
●	Expression of concern about the patient’s level of alcohol consumption
●	Personalized feedback linking alcohol use and health
●	Linkage of alcohol to *admitting* diagnosis, if applicable
●	Advice to abstain/reduce alcohol consumption to below NIAAA limits
●	Assessment of motivation/readiness to change drinking behavior
●	Support for choosing an alcohol consumption goal
●	Strategies/encouragement for reaching alcohol consumption goal (change plan)
●	Potential referral to specialty care or additional support
**Part II: In-hospital follow-up session (12–24 hours after core brief intervention, ~15 minutes)**	
●	Basic reinforcement of core brief intervention
●	Re-assessment and support of motivation and review of change plan (if applicable)
●	Discussion of challenges/barriers to change
●	Consideration of additional strategies for change plan
●	Support and encouragement for reaching goal
**Part III: Telephone follow-up session (2 weeks post-initial brief intervention, ~15 minutes)**	
●	Same as part II

### Arms 2 & 3: Control conditions

Arm 2 (AC) and Arm 3 (AC-LA) comprise the control conditions. Patients in Arms 2 and 3 receive healthy lifestyle brochures that address general healthy lifestyle behaviors such as dietary recommendations, tobacco cessation, and weight management. Brochures were deliberately selected that do not contain content similar to that of the alcohol BI. To ensure parallel attention and number of contacts across groups [41], patients in Arms 2 and 3 also receive a 5–15 minute follow-up telephone call at 2 weeks post-discharge to inquire about general well-being and to review brochure content. Arms 2 and 3 serve as “usual care” insofar as alcohol screening or intervention for hazardous drinking is not routine standard of care for hospitalized patients at this VA medical center.

### Baseline assessment

The extent of patients’ baseline assessments depends on their randomization assignment (Table 2). As previously mentioned, all alcohol consumption data related to the primary outcomes are gathered by the RA during eligibility screening. All sociodemographic/clinical data are gathered immediately prior to randomization. Participants in Arms 1 and 2 are administered the Stages of Change Readiness and Treatment Eagerness Scale (SOCRATES) and the Short Inventory of Problems (SIP-2R) instruments by the RA. The SOCRATES, Version 8, is a 19-item instrument with three subscales: Recognition (of a potential alcohol problem), Ambivalence (about one’s drinking), and Taking Steps (towards behavior change) [42]. Normative data are available to aid in subscale score interpretation. We assess adverse consequences of alcohol use with the SIP-2R, a widely used 15-item, standalone, short version of the Drinker Inventory of Consequences scale [43]. This instrument assesses adverse consequences of alcohol use over the past 3 months in five areas: interpersonal, physical, social, impulsive, and intrapersonal [43]. The SOCRATES and SIP-2R instruments are not administered to Arm 3 participants to reduce the possible impact of AR. Patients randomized to the Arm 3 condition receive only two single items from the SOCRATES and SIP-2R questionnaires that are embedded within the demographic/clinical questionnaire for all participants; this allows for a coarse measure of these constructs in Arm 3 patients.

### Follow-up assessment at 6 months

At 6 months post-discharge, all participants receive the following assessments from the RA by telephone: the sociodemographic questionnaire with embedded general health questions; the AUDIT-C; NIAAA questions regarding the number of drinks consumed per week and number of binge drinking episodes in the past 30 days; the SOCRATES; and the SIP-2R (see follow-up column, Table 2).

### Assessment of intervention fidelity (Specific aim 2)

We are assessing the five classic components of intervention fidelity: quality of delivery, exposure, adherence, participant responsiveness, and program differentiation [44]. These components are defined as follows: (1) quality of delivery: the extent to which all six FRAMES counseling components (feedback, responsibility, advice to change, menu of options, empathy, self-efficacy) [45] and three specific motivational interviewing (MI) techniques (reflective listening, decisional balance, assessment of readiness) [45] are included in the intervention; (2) exposure: the duration of the intervention (minutes); (3) adherence: the number and character of procedural deviations and interruptions to intervention delivery; (4) participant responsiveness: patient opinions about the intervention; and (5) program differentiation: the extent to which only the BI group receives alcohol-related feedback and counseling (i.e., avoiding “cross-contamination” across conditions).

Intervention fidelity is assessed for Parts I and II of the intervention using several data collection methods. First, we are audio recording 100 percent of the Part I interventions. The Study Coordinator will review 50 percent of the audio recordings (randomly selected and stratified by unit over time) to assess quality of delivery, exposure, and adherence. Barriers to intervention fidelity are assessed using several data collection methods. First, the nurse interventionist maintains narrative field notes on each intervention session, focusing on facilitators and barriers to intervention delivery and patient response to the intervention. Quality of delivery is rated using modified versions of established checklists for BI, which are based on the FRAMES model and principles of MI [45-47], upon which BI is based. For the recorded interventions and follow-up calls, we compare the interventionist’s notes to the content of the phone calls. Participant responsiveness is assessed at the conclusion of each intervention. Participants indicate their level of agreement with four statements addressing perceptions of the intervention (e.g., helpfulness, supportiveness) using a 7-point Likert-style scale, and briefly explain each answer in a free-text format below each item. Program differentiation is assessed by asking all groups at the 2-week follow-up call and the 6-month timepoint to recall the extent to which they received any additional alcohol-related feedback, recommendations, or counseling (e.g., primary care provider). These questions are embedded in the sociodemographic/clinical form.

### Nurse interventionist training

Prior to the study, the nurse interventionist, a member of the research team, received a 2-day intensive MI training led by certified trainers from the Motivational Interviewing Network of Trainers [48]. The Principal Investigator provided additional training over the course of one month that was specific to the use of BI techniques. This training included additional role-plays featuring inpatient care scenarios with staff serving as “standardized patients”, as well as training DVDs and webinars demonstrating effective BI patient interactions.

### Analysis plan

For Specific Aim 1, data analysis will focus on the change in alcohol consumption variables from baseline to 6 months across the three arms and, to the extent possible, exploratory analyses of moderators and mediators of the observed effects. The primary independent variable is the randomized treatment group. The primary outcomes of interest are: alcohol screening status, number of drinks/week (past 30 days), and number of binge drinking episodes (past 30 days). Additional outcomes include readiness to change drinking behavior and number of adverse consequences of alcohol use (for patients in Arms 1 and 2 only). Specific Aim 1 will be tested with mixed-effects regression models that account for within-patient correlation and clustering of patients within medical units. To further elucidate the impact of BI on the outcomes of interest, additional post-hoc tests for all pair-wise comparisons (i.e., BI vs. AC, BI vs. AC-LA, AC vs. AC-LA) will be conducted.

To the extent possible, we will also conduct exploratory analyses of potential moderators and mediators of effects due to the intervention. Comparisons between Arm 1 and Arm 2 can include more variables related to the underlying positive changes that BI is designed to facilitate. In particular, we will be able to explore whether the baseline SOCRATES and SIP are important moderators of change in drinking behaviors, in addition to the demographic moderators.

A sample size of 320 that complete the study (160 BI, 80 Attention Control, 80 Attention Control, Limited Assessment) will allow us to detect mean differences of 2.6 drinks per week and 0.34 points on the AUDIT-C. Sample size estimates included expected accrual of veteran inpatients into the study, duration of study participation (6 months), and number of patients expected to be lost to follow-up based on previous BI studies of hospitalized patients (20–30%) [12,14,16,40].

To characterize barriers to intervention fidelity (Specific Aim 2), we will use basic descriptive statistics (e.g., frequency counts, means, medians) to describe the numbers of deviations and interruptions to intervention delivery as planned. Based on the interventionist field notes, we will categorize the types of deviations/interruptions. We will also use these descriptive statistics to describe duration of the intervention (in minutes), the presence/absence of each FRAMES and MI technique within the intervention, and the receipt/nonreceipt of additional alcohol feedback, advice, or counseling. We will also categorize the types of additional feedback/counseling received, the provider involved, and the context in which it occurred. Participant responsiveness data (opinions about the intervention) will be analyzed with frequency counts and simple content analysis of free-text comments.

Additionally, to characterize eligibility, recruitment, and retention of participants, we will develop a recruitment funnel to assess: (a) rates of patient eligibility among hospitalized veterans; (b) rates of refusal for screening; and (c) rates of refusal for study enrollment. We will determine the: (d) mean number of telephone attempts to contact participants at 2 weeks and 6 months; and the (e) proportion of successful to unsuccessful telephone contacts at 2 weeks and 6 months. We will also categorize and generate frequency counts of reasons for: (f) patient ineligibility; (g) screening refusal, and (h) enrollment refusal.

## Discussion

Hospitalization can be a window of opportunity in which to engage the patient who is drinking at hazardous levels in a BI discussion, linking alcohol use to acute/chronic health problems, and then assessing and supporting personal motivation to change drinking behavior, particularly if the hospital admission is alcohol related. This preventative, public health-oriented approach is designed to help avert the physical and psychosocial consequences of hazardous alcohol consumption for individuals and their families. Based on this rationale, and on evidence of the efficacy of BI in other health-care settings, hospital accreditation and quality assurance bodies in the United States have issued and endorsed performance measures pertaining to alcohol BI for hospitalized patients.

Several features of our trial represent improvements in study design over other BI trials, particularly those with inpatients; these features are discussed in the following sections.

### Attention to potential AR

Our three-arm design allows us to compare the effect of the intervention with that of usual care in the presence or absence of extensive alcohol-related assessment. Unequal changes in the primary dependent variables (number of drinks/week, number of binge drinking episodes, change in alcohol screening status) across the two control groups will suggest the presence of AR. To further reduce potential AR, we are limiting the number, scope, and frequency of assessments [21]. We are using only one instrument each for two of the five dependent variables and are limiting the assessment of alcohol use/hazardous drinking to six items. Also, to minimize AR, we have only one assessment timepoint following baseline. Additionally, normative feedback about drinking behavior to both control group participants is provided within the context of general health maintenance recommendations [21].

### Assessment of intervention fidelity

Assessment of intervention fidelity focuses on factors that impede delivery of the intervention and avoid interventionist drift. In our prior work, inpatient nurses identified numerous factors that could affect intervention fidelity and feasibility (e.g., time, potential interruptions for clinical care, and patient privacy and engagement) [49]. Features of acute care delivery and the inpatient setting, such as frequent need for clinical care/testing and shared patient rooms, may significantly impede BI delivery and compromise intervention integrity [50]. Only one other BI study among hospitalized patients has reported efforts to ensure intervention fidelity [12]. Other negative trials either explicitly report difficulties ensuring intervention fidelity (e.g., intervention duration, techniques) [14], or do not report on intervention fidelity at all [15,16].

### Limitation of the entire sample to hazardous, nondependent drinkers

Hazardous, nondependent drinkers are the population for whom BI was intended and in whom it has the greatest demonstrated efficacy [51,52]. Individuals with alcohol dependence typically require treatment of greater intensity and duration than BI provides, and particular attention to issues of loss of control, tolerance, and withdrawal. Other (null) trials of BI for hospitalized patients have included heavier-drinking and/or dependent individuals [12,14,19]. However, as noted earlier, in one case, when the sample was later restricted to hazardous, nondependent users, effects for reduced alcohol consumption were in fact detected [13]. We consider inclusion of only hazardous drinkers in the sample to be a strength because it eliminates the potential “noise” (and null effects) caused by including individuals in the sample for whom BI is not intended.

This study is not designed to answer questions of BI’s efficacy in other groups of drinkers (e.g., dependent individuals), where evidence for the efficacy of BI is uncertain [51,53]. Nonetheless, we recognize the fact that TJC measures do require BI for all patients with a positive alcohol screening (thus potentially requiring BI for patients who also meet criteria for alcohol dependence). Our study results will not be able to address the efficacy of BI for individuals with an alcohol use disorder.

### Patient-level randomization and unequal allocation of patients to groups in a 2:1:1 ratio

This aspect of study design allows more veterans to be exposed to the expected efficacious intervention and permits a more economically feasible study, because fewer hospital units will be needed than with cluster randomization. Also, each hospital unit has its own unique staff, administration, and culture that could influence the delivery and efficacy of the BI. With our design, unit characteristics are equally distributed across participants.

### A three-part BI discussion that includes two follow-up sessions

Follow-up sessions have been recommended by other researchers to increase the effect and salience of the BI [12,40] and to allow exploration of ways to integrate inpatient BI with ongoing attention to hazardous alcohol use in primary care, post-discharge.

### Additional considerations

As our overarching goal is to inform the debate about the efficacy of alcohol BI for hospitalized individuals in the context of imminent widespread adoption of TJC hospital performance measures, this study was not designed as an effectiveness trial that mirrors the measures’ current “real-life” specifications, particularly since such measures are subject to modification by TJC as well as VA’s own External Peer Review Program. Nonetheless, we recognize that our use of the NIAAA questions to determine study eligibility/need for a BI is slightly different from what is specified in the measures from TJC. Specifically, TJC measures dictate that individuals be screened using a validated screening instrument (such as the AUDIT-C) and that individuals with a positive alcohol screen must then receive a BI [4]. Additional assessment of alcohol consumption is not required to make the determination of need for a BI. In contrast, in the VA Substance Use Disorder Clinical Practice Guidelines, the need for BI is not determined by screening result, but by the results of “additional assessment” (i.e., determination of whether the patient is drinking above the NIAAA limits or drinking despite contraindications) [28].

We nonetheless anticipate that the applicability of our results from this trial will not be severely hampered by these differences in use of the NIAAA questions versus AUDIT-C to determine need for BI. If our study ultimately demonstrates the efficacy of BI for reducing drinking in hospitalized patients, then one could argue that we have generated evidence for the efficacy of BI in a “more severe” patient population of drinkers (i.e., those who exceed the NIAAA limits) and that its efficacy in a “less severe” group (i.e., those who “only” had a positive AUDIT-C screen) would still be in question. However, this assertion would be valid only if the AUDIT-C has a high rate of false negatives (i.e., patients who score negative on the AUDIT-C despite drinking above the NIAAA-specified limits). Importantly, in one recent study of almost 500,000 individuals, only about five percent of men and three percent of women in the study had negative AUDIT-C screens, despite reported heavy episodic drinking (i.e., drinking above daily limits) [54].

## Conclusion

The results of this trial will provide greater justification for or against the use of BI performance measures in the inpatient setting, as well as help inform organizational responses to BI-related hospital accreditation and performance measures from TJC and other entities interested in quality of care. Our intervention fidelity and execution data can also inform the specifications of such measures and their implementation. Administrators and clinicians charged with alcohol-related performance measurement and corresponding BI implementation will require efficacious, practical, and patient-centered BI delivery models.

## Abbreviations

AC: Attention control
AC-LA: Attention control - limited assessment
AR: Assessment reactivity
AUDIT-C: Alcohol use disorders identification test – consumption
BI: Brief intervention
CIDI-SAM: Composite international diagnostic interview, substance abuse module
DSM: Diagnostic and statistical manual of mental disorders
EMR: Electronic medical record
ES: Eligibility screening
FRAMES: Feedback, responsibility, advice to change, menu of options, empathy, self-efficacy
IRB: Institutional review board
MI: Motivational interviewing
MINT: Motivational interviewing network of trainers
NIAAA: National institute on alcohol abuse and alcoholism
OEF: Operation enduring freedom
OIF: Operation Iraqi freedom
PI: Principal investigator
RA: Research assistant
SIP-2R: Short inventory of problems – short version
SOCRATES: Stages of change readiness and treatment eagerness scale
TJC: The joint commission
VA: Veterans affairs
VAPHS: VA Pittsburgh healthcare system
